# Cold-Drawn Wood-Filled Polybutylene Succinate Macro-Fibers as a Reinforcing Material for Concrete

**DOI:** 10.3390/polym17030403

**Published:** 2025-02-03

**Authors:** Jonas Herz, Verena Schusser, Dirk Muscat, Nicole Strübbe

**Affiliations:** Faculty of Engineering Sciences, Rosenheim Technical University of Applied Sciences, Hochschulstraße 1, 83024 Rosenheim, Germany

**Keywords:** fibers, compounding, extrusion, multiple drawing, polybutylene succinate, microcrystalline cellulose, wood particles, concrete

## Abstract

The corrosive behavior of steel reinforcements causes issues in the concrete industry. To overcome this issue, alternative noncorrosive reinforcements such as polymer fibers could be used. However, as environmental protection becomes more important, sustainability must also be considered in the solution. An alternative to polymers based on raw oil is bio-based polymers. This study investigates the suitability of polymer fibers produced from polybutylene succinate together with cellulose and wood fillers as concrete reinforcements. Different mixtures of polybutylene succinate, cellulose, and wood fillers were created, and fibers were produced using a multiple drawing process. The fibers were tested using tensile tests, a single-fiber pull-out test, contact angle measurements, reflected light microscopy, density measurements, and thermogravimetric analysis. The fillers were shown to decrease the mechanical properties as the particle size and filler amount increased, resulting in a reduction in Young’s modulus and tensile strength of 55% and 70%, respectively, while adhesion to concrete increased with particle size from 0.31 ± 0.02 N/mm^2^ without filler to 0.90 ± 0.10 N/mm^2^ for the best-performing material combination. Reflected light microscopy images show changes in the fiber surface before and after pull-out. The fiber density decreased from 1.26 ± 0.05 g/cm^3^ to 0.91 ± 0.04 g/cm^3^ with an increasing filler amount and particle size for a compound with 10 weight percent of wood filler 1. The fiber thermal stability decreased slightly with the addition of filler. The greatest effect was a reduction in the temperature to ≈58 °C at 1% weight loss when 10 weight percent of wood was added. This study proves the possibility of using bio-based materials as concrete reinforcements.

## 1. Introduction

Concrete plays an important role in today’s world. After water, it is the second most consumed material [1,2]. Furthermore, today, concrete is the most commonly used material in the building industry [3]. Concrete consists of cement, water, aggregates, and, in many cases, other additives [4]. The required production of cement reached a volume of over 4 billion tonnes in 2013 and has remained at that level since then [5].

Concrete has good compressive strength but a low tensile strength [6]. This makes reinforcement necessary to improve its tensile properties. Reinforcement is commonly provided by steel bars [7], but steel fibers are also used to improve the tensile behavior of concrete [8]. However, the use of steel as a reinforcing material has several disadvantages. The welding of steel bars causes health risks for workers [9]. Another disadvantage results from the corrosive nature of steel; the chloride ion- and carbonate-induced corrosion of steel bars weakens concrete structures through rust formation, followed by cracking and spalling [10,11,12]. This deterioration leads to increasing maintenance costs [13].

The disadvantages of steel have led to research on alternative reinforcing materials. The use of non-metallic reinforcing materials eliminates concerns about carbonation and chloride-induced corrosion [14]. Different organic and inorganic materials, such as polymer fibers [15,16], natural fibers [17,18], glass fibers [19], fiber-reinforced polymers [20], and textile fabrics [21,22], have been investigated. These materials have also shown huge potential for lightweight structures, as the density of these materials, i.e., 2.7 g/cm^3^ for glass [23] and 0.895–0.920 g/cm^3^ for polypropylene (PP) [4] fibers, is much lower compared to that of steel (7.85 g/cm^3^ [24]). The properties of polymeric fibers depend on the polymer used. PP fibers, for example, exhibit a very non-corrosive behavior because of the chemical inertness of polypropylene [16]. However, this results in limited bonding between the fibers and concrete [16,25]. In contrast, polyesters like polyethylene terephthalate (PET) interact with the alkaline environment in concrete, which leads to the partial degradation of the PET fibers and a resulting increase in fiber surface roughness [26]. Sigrüner et al. observed good results for PET fibers in single-fiber pull-out tests (SFPTs) from concrete compared to other polymeric materials [15], indicating a high bonding ability between PET fibers and concrete.

In addition to the properties of the polymer material used, the fiber’s bonding ability is also influenced by other parameters like its shape and surface. Wölfel et al. believed that trilobal-shaped PP fibers featured better anchoring compared to round-shaped ones due to their higher surface area and more complex cross-sectional shape [16]. Fiber surfaces are optimized using different techniques such as fibrillation, crimping, and indentation. All three methods create undercut structures, which causes interlocking between the fibers and the matrix. This mechanical anchoring withstands fiber pull-out from the matrix. Accordingly, several researchers have observed a higher bonding ability between fibers and concrete, as well as a better reinforcement effect, for surface-modified polymer fibers [27,28]. Fiber surface roughness also acts as an undercut structure and creates interlocks, as described by Wölfel et al. [16]. Therefore, it plays a major role in the mechanical anchoring of polymer fibers in concrete [15]. Adding filler particles to the polymer matrix is another method used to increase the surface roughness of polymer fibers. Popa et al. observed increased fiber roughness with the addition of Al_2_O_3_ and CaCO_3_ particles to the outer shell of bicomponent PP fibers [29].

As previously mentioned, many different factors have to be considered when selecting a suitable material for polymer fiber-reinforced concrete. However, sustainability must also be considered. In the future, environmental protection will be needed because of the damage caused by plastic waste in nature. Further, oil-based raw materials might run out, making alternatives necessary. One solution for these two issues could be recycled polymer fibers [30]. Research on recycled PET fibers has already been conducted [26,31,32,33]. Another option could be the use of renewable materials to reduce or avoid the utilization of finite fossil resources [34].

Polybutylene succinate (PBS) is a biodegradable polymer with mechanical properties similar to those of polyolefins [35,36,37]. It is synthesized through the polycondensation of the monomers succinic acid and 1,4-butanediol, which can be obtained from either fossil-based or renewable resources [36,38]. PBS provides excellent processability in textile and extrusion products [37] and is used in many applications such as food packaging [39,40], agricultural films [37,41], medical applications [42], and biocomposites [43,44]. Biocomposites are created using a biodegradable polymer as the matrix and biofibers (e.g., flax or hemp) as the reinforcement material [45]. Biofiber reinforcement enhances specific properties of the whole biocomposite.

Another option for influencing polymer properties, besides using fillers as reinforcement, is changing the molecular orientation of the polymer chains [46]. The molecular orientation can be modified by applying a drawing process. Different methods can be used to carry out polymer drawing. For example, the literature describes discontinuous drawing methods, which involve applying a weight to one end of the fiber and hanging it in a heated furnace [47] or drawing a fiber in a tensile testing machine at a fixed speed [48]. Other methods use two rolls or groups of rolls with different speeds. Sigrüner et al. described a continuous in-line process that takes up the extruded strand and transfers it directly into the heated furnace of the drawing unit [15]. A disadvantage of this method is the relatively high speed of the rolls, which take up the strand from the extrusion and deliver it into the furnace. This results in a high speed on the complete drawing line and an appropriately short residence time in the furnace. Alternative semi-continuous processes decouple the strand extrusion from the later drawing. First, a strand is produced, and it is drawn in a separate step. The drawing can be applied either in a single- [49] or multi-step process [50,51]. All continuous and semi-continuous drawing methods can be executed at room temperature or in a heated furnace. Further, it is also possible to use heated liquids instead of hot air, e.g., a heated glycerol bath [52], to heat the strand. All drawing processes lead to a change in the polymer crystalline structure. A high material stiffness can be achieved if the crystalline structure is oriented high enough [46]. Combining the filler addition and polymer drawing methods allows us to influence selected properties of the resulting fibers, as shown by Chantrasakul et al. [52], Coppola et al. [48], and Pérez et al. [53].

There are no studies on the use of biocomposite fibers as concrete reinforcement. Therefore, this study tried to close the gap by evaluating different cold-drawn wood-filled PBS fibers as concrete reinforcement materials. Different fiber properties, such as mechanical properties and the bonding ability between fiber and concrete, were examined using tensile and single-fiber pull-out tests. The bonding behavior was further evaluated using contact angle measurements and and by conducting reflected light microscopy (RLM) on the fiber surfaces before and after pull-out. Furthermore, the density of the different fibers was tested and compared with that of other reinforcement fibers, and the thermal stability was evaluated using thermogravimetric analysis to compare the influence of different fillers on the combustion behavior.

## 2. Materials and Methods

### 2.1. Materials

**Figure 1:**
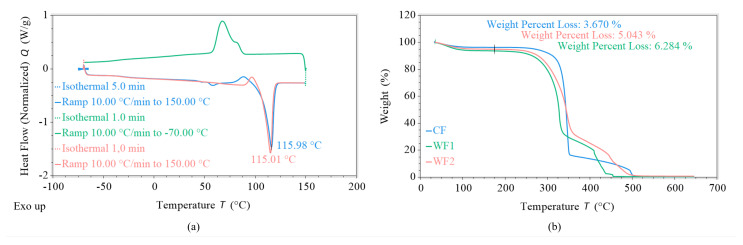
Analysis of raw materials: (**a**) DSC of the used PBS; (**b**) TGAs of the used fillers.

This study used PBS with a melt flow rate (MFR) of 5 g/10 min at 190 °C and 2.16 kg and a density of 1.26 g/cm^3^, as stated in the producers’ specification [54]. The neat polymer was analyzed using differential scanning calorimetry (DSC) before the experiments. Figure 1a) shows a melting peak temperature of ≈115 °C. Further, one microcrystalline cellulose and two different kinds of wood particles were used as filler. The producers’ specifications state the following average particle sizes: microcrystalline cellulose filler (CF), between 6 and 12 µm; wood filler 1 (WF1), between 40 and 70 µm; and wood filler 2 (WF2), between 70 and 150 µm [55,56,57]. All three fillers were analyzed via thermogravimetric analysis (TGA) to evaluate their thermal resistance. For all three filler sizes, Figure 1b) shows a first loss of weight between the starting temperature and 175 °C, due to the water evaporation of the undried material, and thermal combustion is observed to occur between 250 °C and 500 °C.

SFPT specimens were produced using the concrete mixture shown in Table 1. The dry additives were CEN Standard sand [58] and limestone powder, and Portland cement (CEM I 52.5 N) was used as a hydraulic binder. Finally, workability and viscosity were controlled with two superplaticizers, and the water used was obtained from a local tap water system.

### 2.2. Processing

#### 2.2.1. Compounding

Different material combinations of PBS and CF or WF were examined in this study. Table 2 explains the mixing ratios and the resulting material denotations. The neat material PBS-100 was excluded from compounding, while PBS-100-C was passed through a compounder to evaluate the influence of compounding on the material properties. All materials were predried for 10 h at 80 °C in a vacuum furnace. The different material combinations were mixed using a Coperion ZSK MC^18^ compounder with a l/d-ratio of 48. The screw speed was set at 250 rpm, and the throughput was fixed at 8 kg/h. The resulting mass temperature reached values between 159 °C and 169 °C, and the pressure varied between 22 bar and 25 bar depending on the material combination. The residence time of the PBS was 80 s from the mainfeeder, and the filler’s residence time was 60 s from the sidefeeder.

#### 2.2.2. Fiber Production

**Figure 2:**
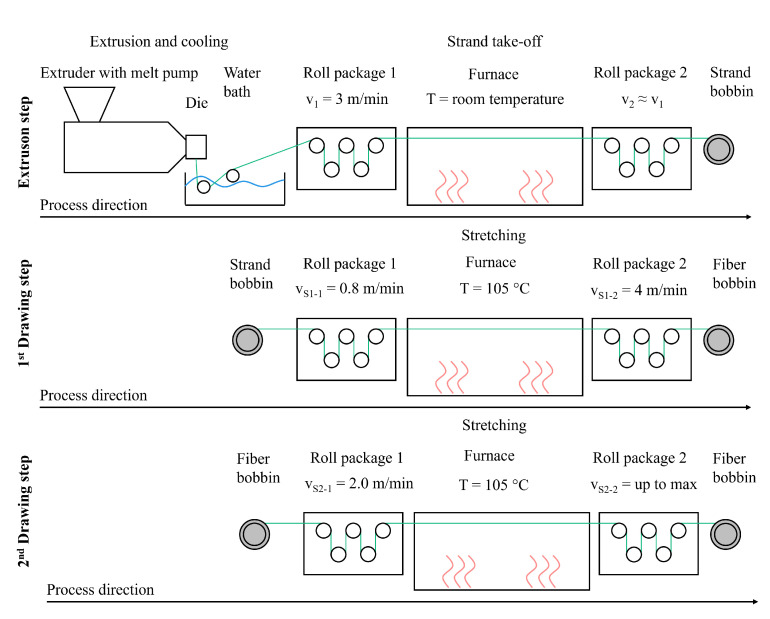
Three-step fiber production process.

Fibers were produced using the multiple drawing method in three steps (Figure 2). First, a strand was produced from neat material and different compounds using a Haake polydrive single-screw extruder equipped with a melt pump. The melt pump was used to fix the throughput at 0.5 kg/h for every material. Extrusion took place at a mass temperature of 153 °C and a die pressure of 19 bar. The extruded strand was cooled in a tempered water bath at ≈38 °C and removed with a roll package with a roll speed of 3 m/min. The undrawn strand was rolled up in a bobbin. Second, the strand was drawn for the first time using a Collin Teach Line MDO stretching unit. Therefore, the bobbin with the strand was placed in front of the stretching unit. Owing to the DSC measurements carried out previously, the furnace was heated up at a temperature of 105 °C; Dahlmann et al. recommend a drawing temperature of 10–20 K below the melting temperature of the polymer used [46]. The strand was taken off the bobbin with the first roll package with a speed of 0.8 m/min and placed in the furnace. Stretching took place because of the higher speed of 4 m/min of the second roll package behind the furnace. The fiber was then rolled up in a bobbin again. Third, a second drawing step was executed by repeating the steps for the first drawing step. The speed of the first roll package was set to 2.0 m/min to avoid large speed differences between the first and second roll package. The speed of the second roll package was varied in steps up to the maximum draw ratio. This study calculated two different draw ratios: the mechanical draw ratio (MDR) and the geometrical draw ratio (GDR). The mechanical draw ratio is defined as follows:

(1) $$MDR=\frac{v_{S1-2}}{v_{S1-1}}\cdot \frac{v_{S2-2}}{v_{S2-1}}$$

where $v_{S1-1}$ and $v_{S1-2}$ are the speeds of the first and second roll packages in the first drawing step, and $v_{S2-1}$ and $v_{S2-2}$ are those in the second drawing step.

The geometrical draw ratio is defined as follows:

(2) $$GDR=\frac{A_{0}}{A_{1}}\cdot \frac{A_{1}}{A_{2}}$$

where $A_{0}$ denotes the cross-sectional areas of the filament before drawing, and $A_{1}$ and $A_{2}$ are the cross-sectional areas of the filament after the first and second drawing step. As the filaments have an elliptical shape, the cross-sectional area is calculated as follows [59]:

(3) $$A=a_{e}\cdot b_{e}\cdot \pi$$

where $a_{e}$ and $b_{e}$ are the major and minor axes of the ellipse, respectively. The filament diameters were measured at both elliptical axes at three points per filament, and the GDR was calculated using the average values.

### 2.3. Analyses

#### 2.3.1. Tensile Tests

Tensile tests were used to evaluate the mechanical properties of the different fibers. The tests were carried out using a tensile test machine (Zwick Roell Z100, Mammelzen, Germany) equipped with a 10 kN load cell, 90° pneumatic deflection grips, and an extensometer for measuring elongation. The test speed was set according to DIN EN 14889-2 at 10 mm/min [60], and Young’s modulus was evaluated according to DIN EN ISO 527-1 between 0.05% and 0.25% elongation [61] with a test speed of 1 mm/min. The tensile strength was calculated using the fibers’ cross-sectional area prior to testing. The cross-sectional area was calculated again as shown in Equation (3).

Five measurements were carried out for each mechanical draw ratio of the different fiber materials. Based on the five measurements, the average values for Young’s modulus and tensile strength were evaluated.

The stress-strain curves for all five measurements per material combination and draw rato are also presented in the Supplementary Files (S1–S13).

#### 2.3.2. Single-Fiber Pull-Out Test

The bonding ability between the polymer fiber and concrete was examined using SFPTs. In this study, the fibers were embedded in the middle of a concrete block with a size of 60 × 60 × 30 mm^3^. The used embedment length of the fibers was 15 mm. The fibers were cleaned using ethanol and fixed in the molds. Then, these were filled with the concrete mixture presented in Table 1, and the concrete was degassed via vibration. The specimens were stored in a climate chamber at 20 °C and 67% humidity for one day and then demolded. Then, these specimen were stored under water in the same climate chamber for 13 additional days.

**Figure 3:**
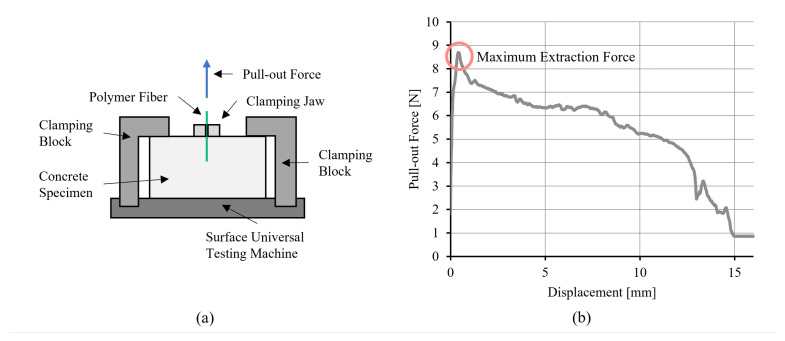
(**a**) Schematic view of SFPT and (**b**) force–displacement diagram.

After 14 days, an SFPT was carried out using a tensile testing machine (Zwick Roell UPM1485, Mammelzen, Germany) equipped with a 500 N load cell. The specimens were fixed on the bottom of the testing machine on four points to prevent moving (Figure 3a). The fiber was clamped with a clamping jaw directly on top of the concrete to avoid a free fiber length that would influence the pull-out result [62]. For testing, the fiber was first preloaded with 2 N and then pulled out with a constant speed of 2 mm/min until complete fiber extraction. The force was detected over the complete pull-out length. Six measurements were carried out for all fiber materials, always with the maximum MDR.

The interfacial shear strength (IFSS) can be calculated from force–displacement curves. The common equation for the IFSS is the following [63]:

(4) $$\tau_{IFSS}=\frac{F_{max}}{A}$$

where $\tau_{IFSS}$ is the IFSS, $F_{max}$ is the maximum extraction force, and *A* is the fiber’s embedded lateral surface.

Because of their elliptical shape, the fibers’ embedded lateral surface is calculated as follows:

(5) $$A=c_{f}\cdot l_{e}$$

where $l_{e}$ is the embedment length of the fiber and $c_{f}$ is the fiber’s circumference, which can be approximately calculated for elliptical shapes with the following equation [59]:

(6) $$c_{f}\approx \pi \left[1,5\left(a_{e}+b_{e}\right)-\sqrt{a_{e}\cdot b_{e}}\right]$$

where $a_{e}$ and $b_{e}$ describe the major and minor axes of the ellipse.

#### 2.3.3. Contact Angle Measurements

The surface energy of the different fiber materials was determined at the maximum draw ratio with contact angle measurements using a KRÜSS EasyDrop FM40 (Bridge Tronic Global, Irvine, CA, USA). The fluids used in the test were water and diodomethane. After fiber cleaning and drying, eight small droplets of each liquid were set on the fiber surface. The contact angle was measured, and the surface energy was calculated with the method of Owen, Wendt, Rabel, and Kaelble using Krüss ADVANCE software. The surface energy can be broken down into a dispersive part and a polar part [64,65]. This allowed us to examine possible correlations with the fibers’ bonding behavior.

#### 2.3.4. Reflected Light Microscopy

Reflected light microscopy was carried using on a Zeiss Smartzoom 5 microscope (Zeiss, Oberkochen, Germany) equipped with a PlanApo D 5x/0.3 FWD 30 mm lens at 600× magnification. Images were taken using an extended depth-of-field software routine to achieve sharp fiber surfaces. The fiber’s surface was evaluated before and after pull-out.

#### 2.3.5. Density Measurements

A Mettler-Toledo MS304TS/00 scale equipped with a density kit was used, with demineralized water as liquid for the density measurements. The densities of the different fiber materials were estimated at their maximum draw ratio. Five samples were taken for every fiber. The samples had a weight in air between 15.8 mg and 17.3 mg.

#### 2.3.6. Thermogravimetric Analysis

The thermal combustion of the fiber materials was evaluated using a TGA 5500 from TA Instruments (New Castle, DE, USA). One sample for every material was tested at the maximum draw ratio. The used samples weighed between 13.0 mg and 15.2 mg and were heated under a nitrogen atmosphere with a heating rate of 20 K/min. The mass was recorded as a function over temperature between ≈35 °C and 650 °C.

## 3. Results and Discussion

### 3.1. Fiber Production

All materials were processable, except for the PBS-90-WF2-10 compound. PBS-90-WF2-10 was excluded from the analysis because an MDR of 5—which was the draw ratio of the first drawing step—was not reached due to fiber breakage early during production.

The other material combination attained different maximum MDRs (see Table 3). The highest MDR of 10 was reached by the PBS-100 reference and the compound with 0.5 wt.% of CF. The one-time compounded neat material (PBS-100-C) reached an MDR of 9, and the MDRs of the compounds from 2 wt.% of CF and all compounds of WF1 and WF2 reached values between 6 and 8. The MDRs for WF1 and WF2 were quite similar; the higher the filler amount, the lower the maximum MDR. PBS-100 and PBS-100-C reached the highest GDRs: 6.32 and 6.34, respectively. The influence of the filler amount on the maximum values became even more visible when comparing the normalized GDRs. The fibers containing CF reached higher GDRs with the same filler amount than WF1 and WF2. WF1 reached slightly higher values than WF2 except in the values for 0.5 wt.%.

**Figure 4:**
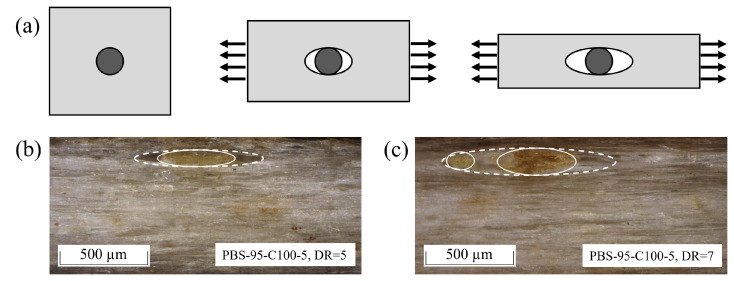
Schematic view (**a**) of filler-caused void formation during polymer drawing and examples for PBS-95-WF2-5 at DR = 5 (**b**) and DR = 7 (**c**) prepared using a microtome. The continuous lines outline the particle, and the dashed lines outline the void.

The differences in the development of the MDR and GDR during drawing are caused by the rigid wood and cellulose fillers. The rigidity of the fillers hinders molecular chain mobility during the stretching process. The lower chain mobility leads to reduced alignment between the molecules, which results in higher filament diameters. This effect is similar to that of reduced elongation at the break of PBS–cotton fiber composites reported by Calabia et al. [66]. The general reduction in draw ratios is caused by the reduced chain mobility and defects in the filament structure such as voids or cavities. These defects result from the drawing of polymer-containing particles, which supports the observations of Rattanawijan and Amornsakchai [67]. The rigid filler particles are not able to deform under drawing stress, which leads to debonding on both sides of the particle—parallel to the drawing stress—and opens a space, as Kim et al. showed in their study on micromechanical deformation [68]. The described effect is shown schematically in Figure 4a, and Figure 4b,c show two examples of void formation caused by wood particles for PBS-95-WF2-5 at DRs of 5 and 7.

### 3.2. Mechanical Properties

Table 3 compares the mechanical properties for all fiber materials at the maximum draw ratio. PBS-100 shows a Young’s modulus of 1836.08 ± 86.10 MPa and a tensile strength of 410.12 ± 7.30 MPa at a maximum MDR of 10. The compounded but unfilled reference (PBS-100-C) reaches a similar Young’s modulus but decreases in tensile strength by ≈25%. This indicates material degradation during the compounding process. The filled materials attain lower mechanical properties than the directly processed PBS-100 and the compounded, unfilled PBS-100-C, except the fiber filled with 0.5 wt.% of CF. For this material, the tensile strength is higher than the compounded reference, but it is still lower than the directly processed PBS-100. In general, the mechanical properties deteriorate with an increase in filler size and amount. This decrease may result from the mentioned structural defects in the fiber caused by the fillers, as no force can be transferred by the voids and partially bounded fillers. The elongation at maximum force reaches values between 14.30 ± 0.99% and 20.18 ± 1.38%. In the case of elongation, there is no significant effect observed with the addition of fillers.

**Figure 5:**
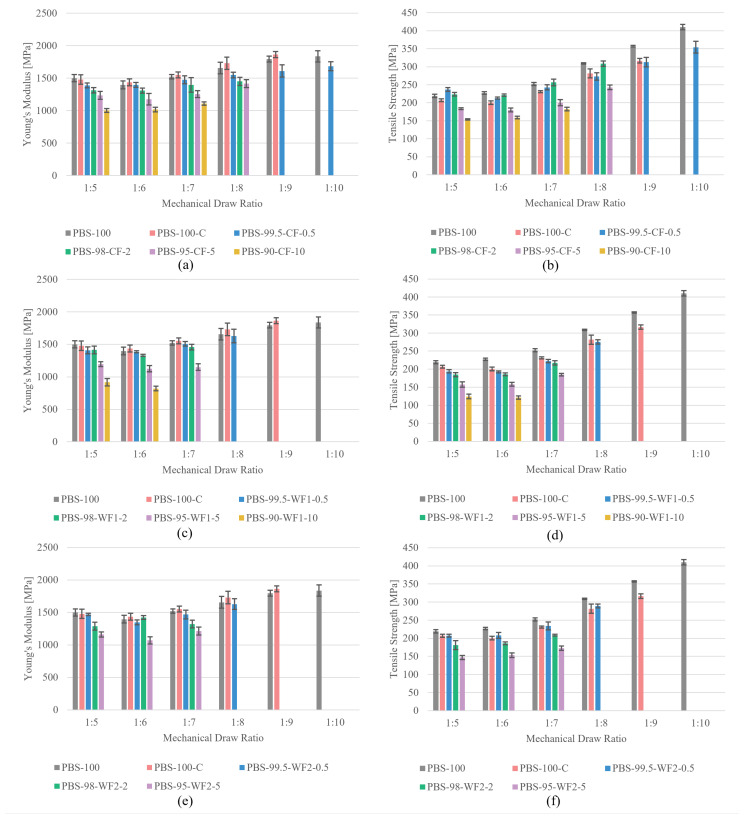
Tensile test results: (**a**) Young’s modulus and (**b**) tensile strength for fibers containing CF; (**c**) Young’s modulus and (**d**) tensile strength for fibers containing WF1; (**e**) Young’s modulus and (**f**) tensile strength for fibers containing WF2.

Figure 5 shows the Young’s modulus and tensile strength plotted against the MDR for all produced materials. Nearly all materials show an enhancement in Young’s modulus and tensile strength with an increasing MDR. Only fibers with higher amounts (5 or 10 wt.%) of WF1 or WF2 show no increase in Young’s modulus. For the tensile strength, improvement stops at a filler amount of 10 wt.% of WF1. All fillers cause a decrease in the mechanical properties with an increase in the filler amount. The only exception is the effect of CF on the tensile strength. CF in amounts of 0.5 and 2 wt.% achieves tensile strengths that are higher than those of both unfilled reference materials at an MDR of 5. At an MDR of 6, their tensile strengths are between those of the PBS-100 and PBS-100-C references. PBS-99.5-CF-0.5 is still between the references at an MDR of 7, while PBS-98-CF-2 maintains the tensile strength from PBS-100. For an MDR of 8, PBS-100 and PBS-98-CF-2 also reach similar tensile strengths. The tensile strength of PBS-99.5-CF-0.5 is slightly lower than that of PBS-100-C, and the same result is visible at an MDR of 9, for which the tensile strength of PBS-100-C is slightly higher than that of PBS-99.5-CF-0.5. It seems that there is a slight reinforcing effect caused by 0.5 and 2 wt.% of CF, and higher possible draw ratios without fillers seem to modify this effect.

The mechanical properties presented here are relatively low compared to those of polymer fibers for concrete reinforcements presented in the literature. Different papers have presented values of Young’s modulus between 4.3 GPa and 10 GPa and tensile strengths between 400 MPa to 640 MPa for polyolefine and PP macro-fibers [15,69,70,71]. Further, Sigrüner et al. reported a tensile strength of ≈495 MPa and a Young’s modulus of 13.3 GPa for PET fibers [15]. There is a high difference in Young’s modulus, as the values from the literature are more than two to five times higher than those from the PBS fibers in this study, but the difference is lower for tensile strength. PBS-100 reaches lower strength values for polypropylene fibers. However, to achieve good reinforcing effects in concrete, the fibers should surpass the matrix’s Young’s modulus and tensile strength [72,73]. The Young’s modulus of concrete is between 30 GPa and 50 GPa [74]. Accordingly, it is not possible to reach this Young’s modulus with this study’s PBS fibers, or with those PP fibers in the literature mentioned. However, Neunzig et al. presented a uniaxial tensile strength of concrete in the range of 4 to 5 MPa [75]. Correspondingly, the strength values of the PBS fibers satisfy the requirements.

### 3.3. Single-Fiber Pull-Out Test

**Figure 6:**
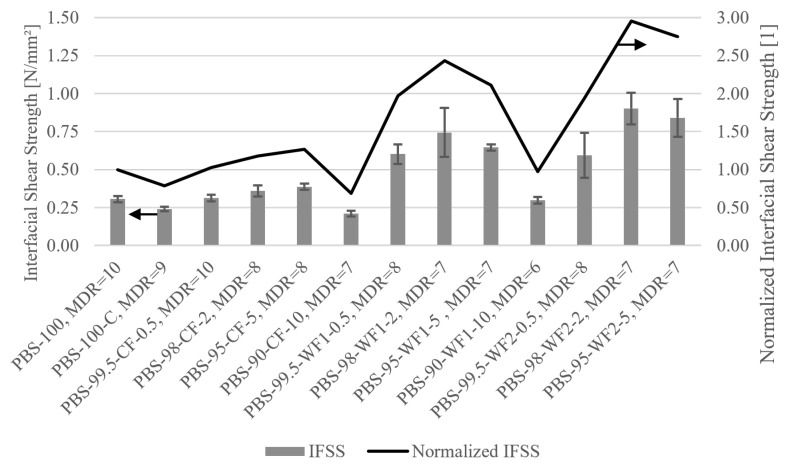
IFSSs and normalized IFSSs for different fiber materials at their maximum MDRs.

Figure 6 shows the IFSS and normalized IFSS (with PBS-100 as a reference) results from carrying out SFPTs on every fiber at its maximum draw ratio. All material mixtures increase or maintain the IFSS of the unfilled fibers, except the materials containing 10 wt.%. A maximum IFSS of 0.9 N/mm^2^ is achieved with PBS-98-WF2-2, which is approximately three times higher than the IFSS of PBS-100. Both WFs achieve the maximum IFSS at a filler amount of 2 wt.%, while the fibers containing CF reach their maximum at a filler amount of 5 wt.%.

Fiber matrix bonding is influenced by several fiber properties, like fiber surface deformation, fiber strength, and fiber elastic modulus [69]. Correspondingly, Sigrüner et al. reported a high correlation between Young’s modulus and IFSS [15]. Comparing the IFSS with the Young’s modulus in Table 3 shows the influence of the fiber mechanics. Firstly, an increase in the IFSS is observed as the amount of filler in the fiber material increases even though Young’s modulus decreases for all fibers containing fillers. However, it seems that the effect of the fillers on the IFSS is larger than that from the fiber mechanics. When Young’s modulus decreases further, the effect of the fillers is surpassed, and the IFSS decreases.

Pitkethly et al. observed that the test setup and analysis method used exert considerable influence on the results [76]; thus, carrying out a quantitative comparison with other SFPT results from the literature is difficult. However, because Sigrüner et al. used a test setup for their SFPT similar to the one used in this research, with only a higher number of test specimens, a quantitative comparison is possible. IFSSs of ≈0.36 N/mm^2^ and ≈0.43 N/mm^2^ were reported for a low-density polyethylene (LDPE) and a high-density polyethylene (HDPE) fiber. The PP fiber reached an IFSS of ≈0.89 N/mm^2^, and the PET fiber, 1.34 N/mm^2^ [15]. None of the PBS fibers reached the IFSS of the PET fiber, but PBS-98-WF2-2 was in the range of the PP fiber. The neat PBS fiber’s IFSS was below that of LDPE, but most IFSSs of CF and WF fibers reached or surpassed the IFSS of the LDPE or HDPE fibers. For other research results, only trends can be transferred because of the differences in the used test setups. However, an increase in the IFSS from the use of fillers in core–shell fibers was reported by Popa et al. [29]. Therefore, fillers open up the opportunity to enhance the ability of polymer fibers to bond with concrete.

### 3.4. Contact Angle Measurements

**Figure 7:**
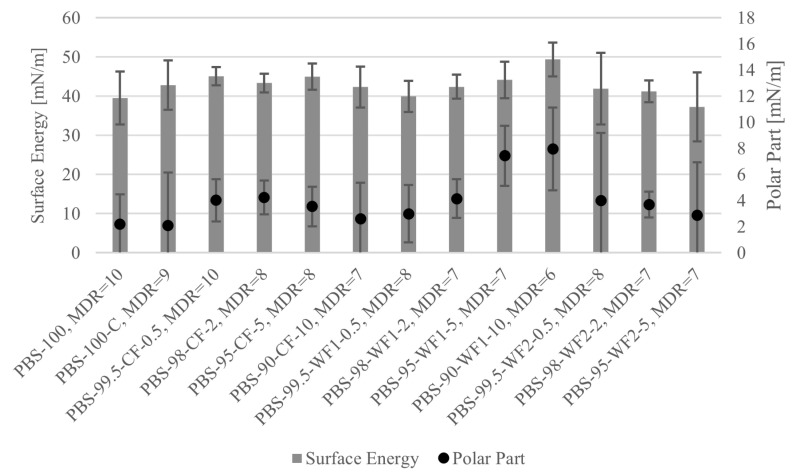
Surface energy and polar part for different fiber materials at their maximum MDRs.

Surface energies and accompanying polar parts of the different fibers are shown in Figure 7. CF slightly increases or maintains the surface energy compared to the unfilled fibers PBS-100 and PBS-100-C. Also, WF1 shows a small increase with higher filler amounts, and WF2 shows no difference with a slight tendency to lower the surface energy with an increasing filler amount. The polar parts show similar behavior. CF-containing fibers reach slightly higher values for 0.5, 2, and 5 wt.% compared to PBS-100 and PBS-100-C, while PBS-90-CF-10 maintains the polar part of the unfilled materials. There is a visible increase in the polar part with an increasing amount of WF1, and WF2-containing fibers show slightly increased values compared to the unfilled ones.

The surface energy of domestic woods measured by other researchers varies between values of ≈47 mN/m and ≈80 mN/m, and the presented polar part reaches values from ≈6 mN/m to ≈30 mN/m, depending on the type of wood [77,78,79,80]. Accordingly, the increase in the surface energy and polarity of the filled fibers can be explained by the larger active surface of filler particles. Further, Wokenhauer et al. and Gindl et al. showed that the surface energy and the polarity decrease via wood aging, which can reduce the surface energy of some wood types to values below 50 mN/m, and their polar part to nearly 0 mN/m [77,79]. The different behaviors of WF1 and WF2 may be caused either by a different mixture of wood types or wood particles with a different age. The high standard deviations might result from the distribution of filler particles on the fiber’s surface. A comparison with the IFSSs from Figure 6 shows that there is no correlation between the IFSS and surface energy/polar part. This leads to the assumption that the bonding behavior is influenced more by other factors like Young’s modulus or surface roughness.

### 3.5. Reflected Light Microscopy

**Figure 8:**
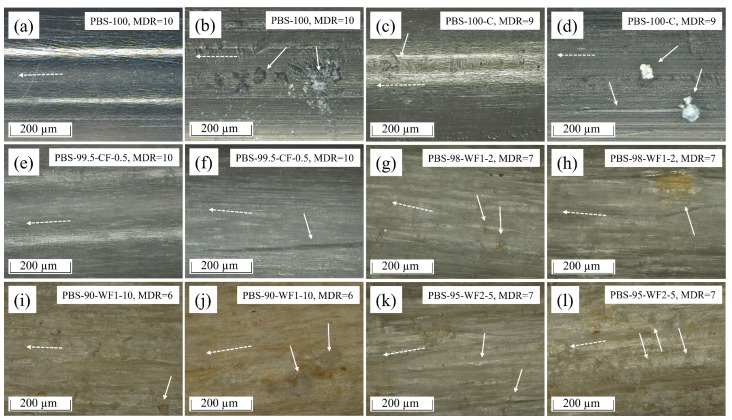
Pictures obtained from reflected light microscopy: (**a**) PBS-100 before pull-out; (**b**) PBS-100 after pull-out with embossments and attached concrete; (**c**) PBS-100-C before pull-out with tiny surface deformation; (**d**) PBS-100-C after pull-out with attached concrete particles and a resulting scratch; (**e**) PBS-99.5-CF-0.5 before pull-out; (**f**) PBS-99.5-CF-0.5 after pull-out with a scratch; (**g**) PBS-98-WF1-2 before pull-out: the semitransparent fiber surface shows wood particles in the matrix and small voids around them; (**h**) PBS-98-WF1-2 after pull-out with scratches and partially uncovered wood particles; (**i**) PBS-90-WF1-10 before pull-out with wood particles on the fiber surface; (**j**) PBS-90-WF1-10 after pull out with adherence to concrete; (**k**) PBS-95-WF2-5 before pull-out with wood particles and voids under semitransparent surface; (**l**) PBS-95-WF2-5 after pull-out with adherence to concrete. (Dashed arrows indicate the fiber direction).

Figure 8 shows pictures obtained by conducting RLM on some of the materials before and after pull-out. There are adhering concrete particles and multiple surface deformations visible for PBS-100 after pull-out (Figure 8b) compared to the fiber before pull-out (Figure 8a). Figure 8d) shows concrete particles and a scratch caused by one of the concrete particles on the PBS-100-C fiber after pull-out. The surface of the PBS-100-C fiber before pull-out shows some small deformations (Figure 8c), and the fiber from PBS-99.5-CF-0.5 (Figure 8e) looks similar to PBS-100. No surface deformation is visible. PBS-99.5-CF-0.5 after pull-out shows a large scratch (Figure 8f). In Figure 8g, the partial transparent surface of the PBS fibers is visible. Due to the semitransparent drawn PBS, some wood particles below the surface of the PBS-98-WF1-2 fiber can be observed. PBS-98-WF1-2 shows scratches and a partially uncovered wood particle after pull-out (Figure 8h). The brown color of PBS-90-WF1-10 in Figure 8i,j is again caused by the partial transparency, which allows us to see the color of the wood particles below the surface. There is an uncovered wood particle on the PBS-90-WF1-10 fiber surface before pull-out on the bottom right of Figure 8i. The brown color increases after pull-out, which indicates that some of the covering PBS is sheared off during pull-out. Further, there is concrete adhering to the surface of PBS-90-WF1-10 after pull-out. PBS-95-WF2-5 in Figure 8k shows another effect. Due to the partial transparency and the larger size of the wood particles, some voids around the wood particles become visible. This supports the explanation for the decreased fiber mechanics due to the delamination of the filler particle during the drawing process. PBS-95-WF2-5 after pull-out (Figure 8l) shows a surface similar to that of PBS-90-WF1-10. The fibers of the materials that are not shown in Figure 8 show similar effects to the ones presented here.

### 3.6. Density Measurements

**Figure 9:**
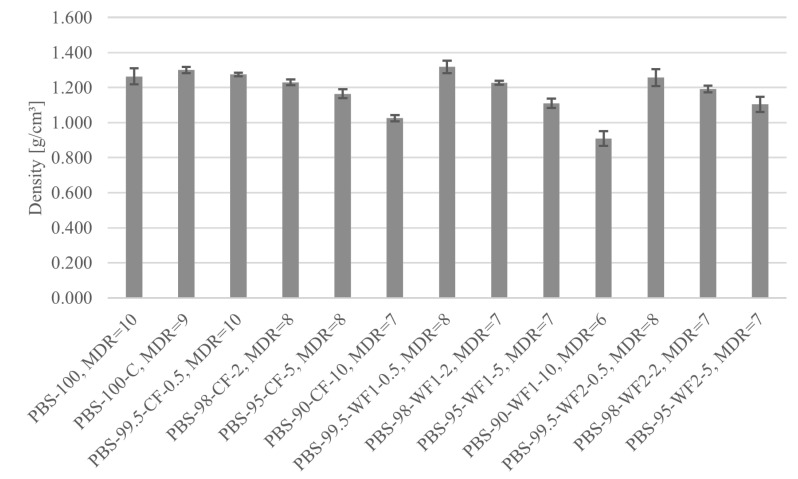
Density for different fiber materials at their maximum MDRs.

The densities of the different fiber materials at their maximum draw ratios are shown in Figure 9. The density of the unfilled fibers PBS-100 and PBS-100-C is between 1.26 g/cm^3^ and 1.3 g/cm^3^. The fibers containing 0.5 wt.% of CF or WF reach densities similar to those of the unfilled materials. With an increasing filler amount, the density decreases for all filler types. The lowest density is reached by PBS-90-WF1-10 with 0.91 g/cm^3^. The density of the used PBS is 1.260 g/cm^3^ [54], and the density of microcrystalline cellulose is ≈1.46 g/cm^3^, as reported by Sun [81]. Considering the higher density of microcrystalline cellulose, the density of the compounded material should increase compared to the density of PBS. The gross density of softwood is reported to be between ≈0.45 g/cm^3^ and ≈0.53 g/cm^3^ [82]. The low density is a result from empty space in the wood vessels [83]. However, Yuan et al. and Obermeier et al. reported that these empty spaces are filled with polymer during compounding [83,84]. If the empty spaces are filled with polymer, only the cell wall structure of the wood remains. The density of the wooden cell walls is ≈1.56 g/cm^3^ according to Kollmann [85]. As the density of the wooden cell wall is higher than the density of PBS, the density of the wood-containing compounds should also increase. The opposite development of the fiber density for fibers containing microcrystalline cellulose and wood fillers can be explained again through the creation of voids in the fiber structure during fiber drawing due to the filler particles. A comparison with the density of steel (7.85 g/cm^3^ [24]) or other glass fibers (2.7 g/cm^3^ [23]) shows the potential application of the lightweight PBS fibers tested.

### 3.7. Thermogravimetric Analysis

**Figure 10:**
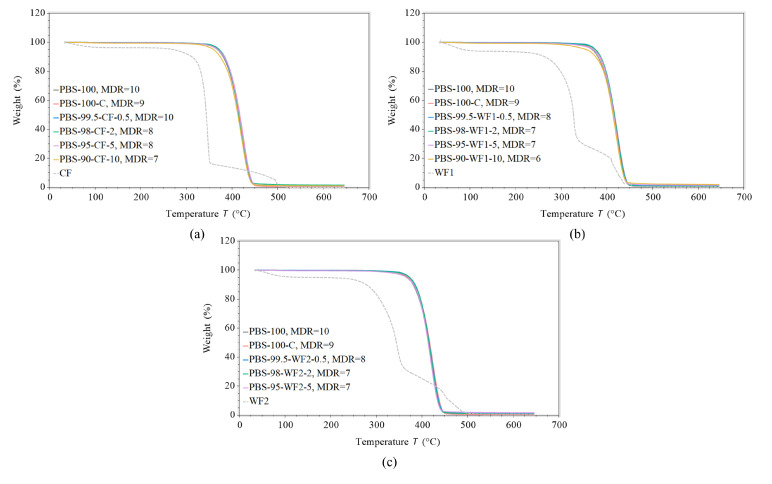
TGA results: weight loss over temperature for fibers containing (**a**) CF, (**b**) WF1, and (**c**) WF2, at their maximum draw ratio. The dashed gray curves show the weight loss of the filler material.

The thermogravimetric analysis results show a shift to lower temperatures as the filler content increases for all filler types (Figure 10). Table 4 summarizes comparative parameters like the onset point, temperatures at 1, 2, and 5% weight losses, and the maximum degradation rate derived from the curves shown in Figure 10.

The onset point does not appear to be a representative parameter, as it varies between 380.06 °C and 387.50 °C for all materials. In addition, the maximum degradation rate is not representative, with values between −2.008 %/°C and −2.294 %/°C. The temperatures at 1, 2, and 5 % weight loss show decreasing temperatures with an increasing filler load for all filler particles. This effect is more distinctive for WF than for CF. CF-containing fibers show a loss in temperature at 1% weight loss from 333.52 °C for 0.5 wt.% to 300.29 for 10 wt.%, which is a difference of 33.23 °C. For WF1-containing fibers, the temperature drops with a delta of 73.98 °C from 342.34 °C for 0.5 wt.% to 268.36 °C for 10 wt.% at 1% weight loss. The temperature differences decrease with an increasing percentage in weight loss. For example, the difference between PBS-99.5-CF-0.5 and PBS-90-CF-10 decreases from 33.23 °C for 1% weight loss to 12.58 °C for 5% weight loss. In the case of WF1, the difference decreases from 73.98 °C for 1% weight loss to 20.34 °C at 5% weight loss.

A comparison with the weight losses of the filler materials, which are represented by the dashed gray curves in Figure 10, shows that the degradation of the fillers without polymer begins before 300 °C, which is much earlier than the onset of degradation of the unfilled PBS-100 and PBS-100-C samples. This explains the shift in the degradation temperature with increasing filler amount. The thermally less stable fillers lower the thermal stability of the fully filled fiber material.

## 4. Conclusions

PBS was compounded with microcrystalline cellulose and two different wood particles. Fibers were produced from the compounds using a multiple drawing method, and the fibers were tested to investigate their suitability as concrete reinforcement. Tensile tests highlight a deterioration in mechanical properties caused by filler addition and the compounding process. On the other hand, the results from single-fiber pull-out tests indicate an increased interfacial shear strength for the filled materials, which, in the case of WF2, is approximately three times higher than that of the unfilled material. Contact angle measurements show only slight influences of the fillers on the surface energy and polarity, and no correlation with the IFSS. Pictures obtained from reflected light microscopy show deformations and evidence that concrete has adhered to the fiber surfaces after pull-out. The microscopy images of filled fibers before pull-out show voids. These may explain the reduced fiber mechanics and decreasing fiber density with increasing filler size and amount. TGA shows that the thermal degradation of fibers filled with higher amounts of microcrystalline cellulose and wood particles occurs earlier.

This study indicates that concrete reinforcement with bio-based polymer fibers is theoretically possible. Due to the low fiber density, the use of these fibers in lightweight structures can be considered a lightweight reinforcement that lowers the weight of the entire concrete element. On the other hand, the low Young’s moduli and tensile strengths of the microcrystalline cellulose and wood-filled PBS fibers may be too low to achieve real structural reinforcement. Another application for the filled PBS fibers could be the prevention of early crack propagation during concrete curing, as the stress caused by shrinkage should be comparatively low. Further research would be necessary to evaluate the real reinforcement potential of PBS fibers and their use in possible applications. Accordingly, the next step could be the evaluation of the reinforcement efficiency of the PBS fibers presented here in more realistic test setups. In particular, compressive, tensile, and flexural tests using fiber-reinforced concrete with different formulations and at different fiber loadings are of interest.

## Figures and Tables

**Table 1:** Concrete mixture.

Material		Sand	CEM I 52.5 N	Limestone Powder	Water	Superplasticizer	
						**1**	**2**
Amount	(g)	1350	450	75	220	6.2	3.0
Amount	(wt.%)	64.15	21.38	3.56	10.45	0.29	0.15

**Table 2:** Denotations and formulations of used fiber materials.

No.	Material Name	PBS Content	CF Content	WF1 Content	WF2 Content
		**[wt.%]**	**[wt.%]**	**[wt.%]**	**[wt.%]**
1	PBS-100	100	-	-	-
2	PBS-100-C	100	-	-	-
3	PBS-99.5-CF-0.5	99.5	0.5	-	-
4	PBS-98-CF-2	98.0	2.0	-	-
5	PBS-95-CF-5	95.0	5.0	-	-
6	PBS-90-CF-10	90.0	10.0	-	-
7	PBS-99.5-WF1-0.5	99.5	-	0.5	-
8	PBS-98-WF1-2	98.0	-	2.0	-
9	PBS-95-WF1-5	95.0	-	5.0	-
10	PBS-90-WF1-10	90.0	-	10.0	-
11	PBS-99.5-WF2-0.5	99.5	-	-	0.5
12	PBS-98-WF2-2	98.0	-	-	2.0
13	PBS-95-WF2-5	95.0	-	-	5.0
14	PBS-90-WF2-10	90.0	-	-	10.0

**Table 3:** Maximum draw ratios and corresponding mechanical properties for every fiber material.

Sample Code	Mechanical Draw Ratio	Geometrical Draw Ratio	Normalized Geometrical Draw Ratio	Young’s Modulus	Tensile Strength	Elongation at Maximum Force
	**[1]**	**[1]**	**[1]**	**[MPa]**	**[MPa]**	**[%]**
PBS-100	10	6.32	1	1836.08 ± 86.10	410.12 ± 7.30	16.01 ± 0.20
PBS-100-C	9	6.34	1	1862.70 ± 45.82	316.66 ± 6.27	14.79 ± 0.14
PBS-99.5-CF-0.5	10	5.99	0.95	1684.43 ± 68.62	354.25 ± 16.06	17.03 ± 0.95
PBS-98-CF-2	8	5.16	0.82	1450.26 ± 62.74	308.30 ± 7.43	17.10 ± 0.40
PBS-95-CF-5	8	4.70	0.74	1415.41 ± 59.86	242.58 ± 6.41	17.27 ± 0.64
PBS-90-CF-10	7	4.06	0.64	1108.67 ± 25.60	182.54 ± 5.09	20.18 ± 1.38
PBS-99.5-WF1-0.5	8	5.30	0.84	1629.01 ± 104.54	275.41 ± 5.81	14.62 ± 0.16
PBS-98-WF1-2	7	5.14	0.81	1456.69 ± 43.53	217.13 ± 6.06	14.74 ± 0.77
PBS-95-WF1-5	7	4.57	0.72	1148.08 ± 51.33	185.09 ± 3.37	14.79 ± 0.50
PBS-90-WF1-10	6	3.41	0.54	820.31 ± 35.97	121.56 ± 4.85	14.30 ± 0.99
PBS-99.5-WF2-0.5	8	5.62	0.89	1627.99 ± 32.65	289.10 ± 5.32	15.19 ± 0.25
PBS-98-WF2-2	7	4.98	0.79	1321.16 ± 59.63	208.79 ± 2.63	15.33 ± 0.57
PBS-95-WF2-5	7	4.39	0.70	1212.77 ± 60.05	172.49 ± 5.94	15.52 ± 0.89

**Table 4:** Onset point, temperatures at 1, 2, and 5 % weight loss, and the maximum degradation rate for every fiber material at its maximum draw ratio.

Sample Code	Mechanical Draw Ratio	Onset Point	Temperature at 1% Weight Loss	Temperature at 2% Weight Loss	Temperature at 5% Weight Loss	Maximum Degradation Rate
	[1]	**[°C]**	**[°C]**	**[°C]**	**[°C]**	**[%/°C]**
PBS-100	10	383.93	326.29	348.37	367.79	−2.011
PBS-100-C	9	386.72	330.15	351.71	371.10	−2.170
PBS-99.5-CF-0.5	10	384.31	333.52	356.91	372.10	−2.078
PBS-98-CF-2	8	385.67	337.59	355.82	371.80	−2.215
PBS-95-CF-5	8	384.39	324.56	347.65	368.63	−2.092
PBS-90-CF-10	7	380.06	300.29	336.02	359.52	−2.008
PBS-99.5-WF1-0.5	8	387.50	342.34	360.88	375.17	−2.138
PBS-98-WF1-2	7	387.13	327.37	356.88	374.91	−2.258
PBS-95-WF1-5	7	384.24	305.56	338.72	370.04	−2.294
PBS-90-WF1-10	6	384.74	268.36	312.94	354.83	−2.076
PBS-99.5-WF2-0.5	8	385.64	338.73	356.76	372.89	−2.130
PBS-98-WF2-2	7	385.56	326.77	354.33	372.93	−2.220
PBS-95-WF2-5	7	386.73	302.47	335.44	366.08	−2.215

## Data Availability

The data presented in this study are available on request from the corresponding author.

## Supplementary Materials

The following supporting information can be downloaded at: https://www.mdpi.com/article/10.3390/polym17030403/s1. Figure S1: Stress-strain curves from tensile tests on PBS-100 at different draw ratios; Figure S2: Stress-strain curves from tensile tests on PBS-100-C at different draw ratios; Figure S3: Stress-strain curves from tensile tests on PBS-99.5-CF-0.5 at different draw ratios; Figure S4: Stress-strain curves from tensile tests on PBS-98-CF-2 at different draw ratios; Figure S5: Stress-strain curves from tensile tests on PBS-95-CF-5 at different draw ratios; Figure S6: Stress-strain curves from tensile tests on PBS-90-CF-10 at different draw ratios; Figure S7: Stress-strain curves from tensile tests on PBS-99.5-WF1-0.5 at different draw ratios; Figure S8: Stress-strain curves from tensile tests on PBS-98-WF1-2 at different draw ratios; Figure S9: Stress-strain curves from tensile tests on PBS-95-WF1-5 at different draw ratios; Figure S10: Stress-strain curves from tensile tests on PBS-90-WF1-10 at different draw ratios; Figure S11: Stress-strain curves from tensile tests on PBS-99.5-WF2-0.5 at different draw ratios; Figure S12: Stress-strain curves from tensile tests on PBS-98-WF2-2 at different draw ratios; Figure S13: Stress-strain curves from tensile tests on PBS-95-WF2-5 at different draw ratios.

## Abbreviations

The following abbreviations are used in this manuscript:

CF	Cellulose Filler;
DSC	Differential Scanning Calorimetry;
GDR	Geometrical Draw Ratio;
IFSS	Interfacial Shear Strength;
HDPE	High-Density Polyethylene;
LDPE	Low-Denisty Polyethylene;
MDR	Mechanical Draw Ratio;
PBS	Polybutylene Succinate;
PET	Polyethylene Terephtalate;
PP	Polypropylene;
RLM	Reflected Light Microscopy;
SFPT	Single-Fiber Pull-Out Test;
TGA	Thermogravimetric Analysis;
WF	Wood Filler.
