# Supplementary figures and images for: Cold-Drawn Wood-Filled Polybutylene Succinate Macro-Fibers as a Reinforcing Material for Concrete
Source: Polymers (Basel). 2025 Feb 3;17(3):403. doi: 10.3390/polym17030403 (PMC11821048; doi:10.3390/polym17030403)

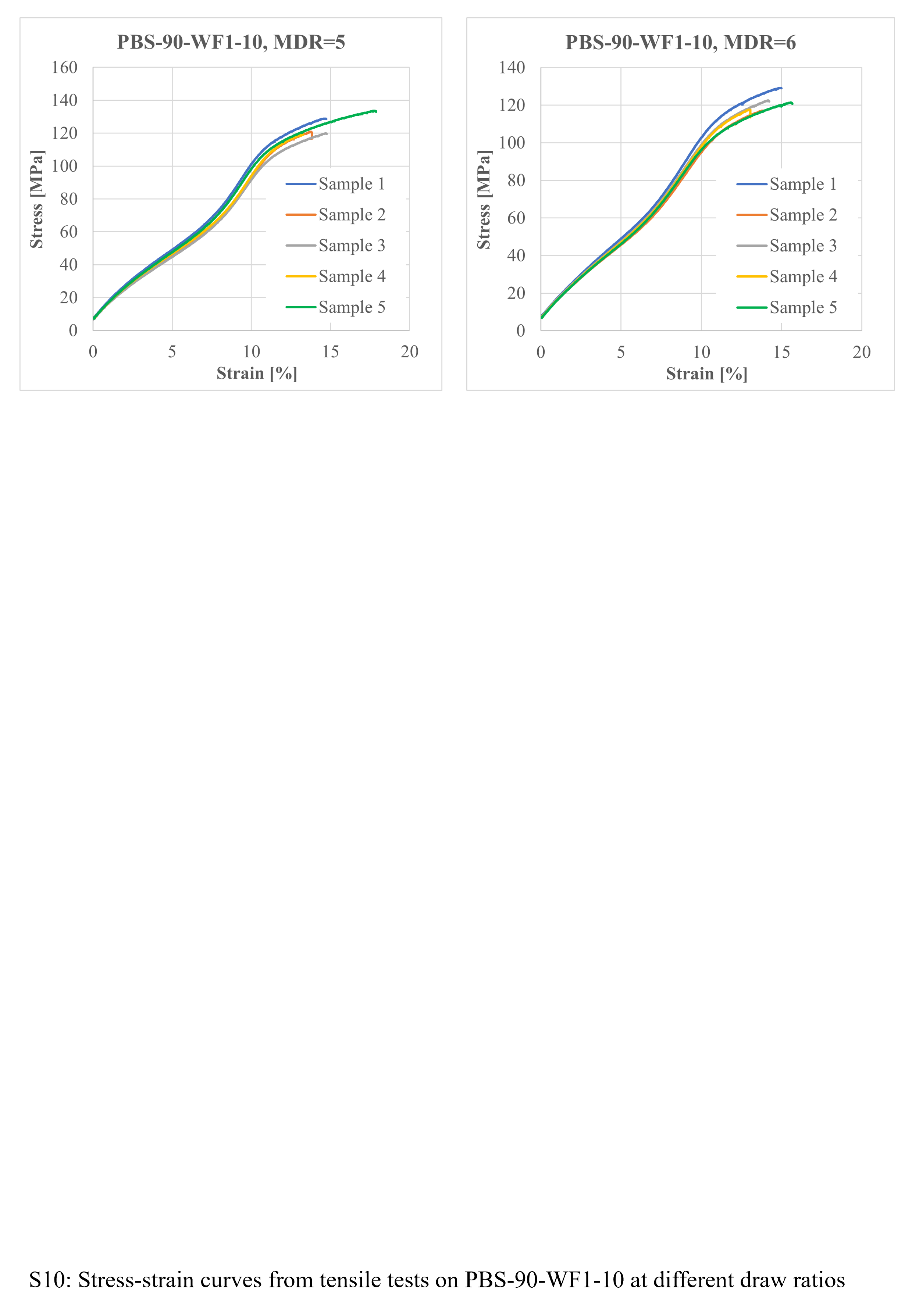

Supplement: Supplementary file 1 [file polymers-17-00403-s001.zip › S10_stress-strain_curves_PBS-90-WF1-10.png]

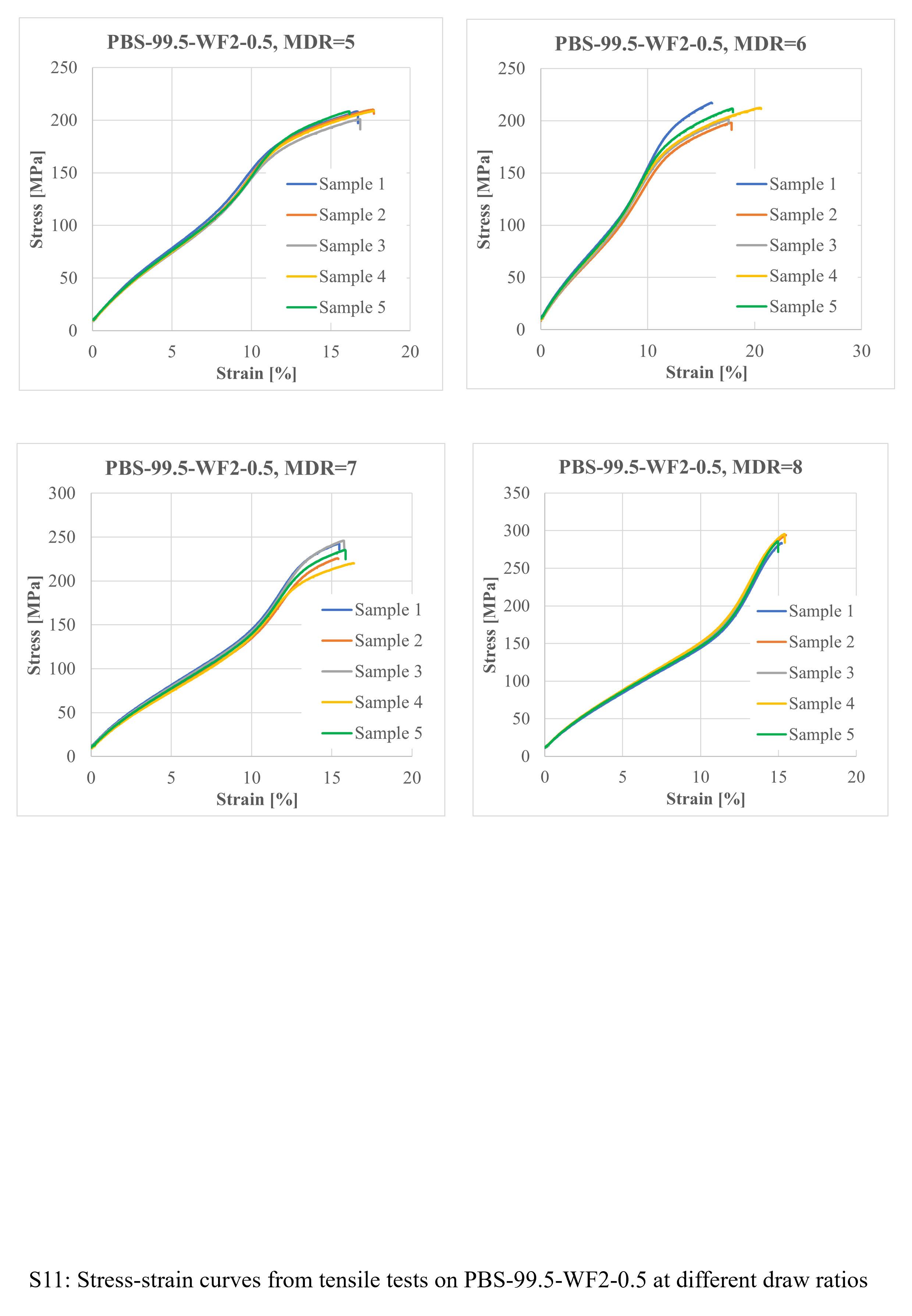

Supplement: Supplementary file 1 [file polymers-17-00403-s001.zip › S11_stress-strain_curves_PBS-99,5-WF2-0,5.png]

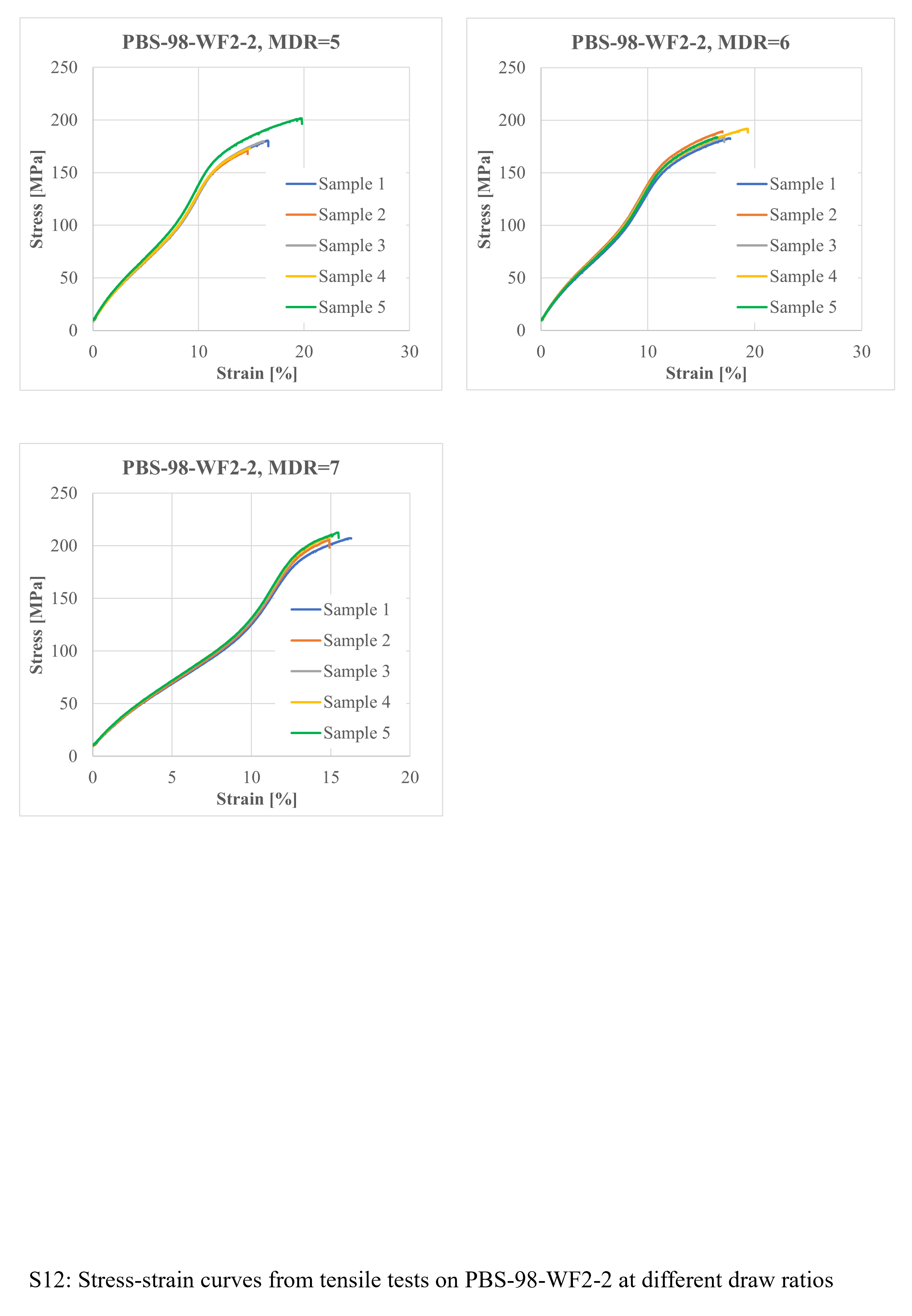

Supplement: Supplementary file 1 [file polymers-17-00403-s001.zip › S12_stress-strain_curves_PBS-98-WF2-2.png]

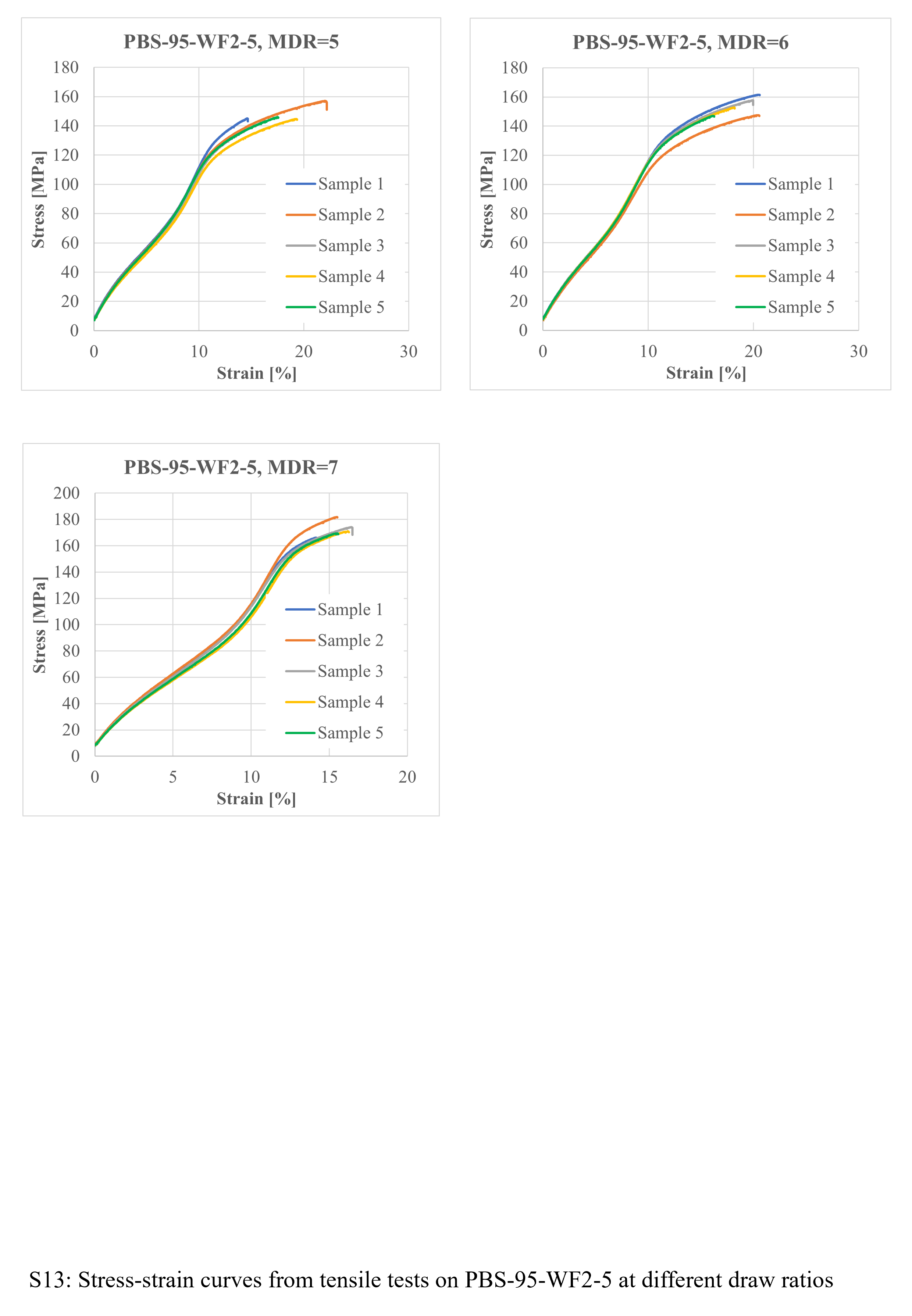

Supplement: Supplementary file 1 [file polymers-17-00403-s001.zip › S13_stress-strain_curves_PBS-95-WF2-5.png]

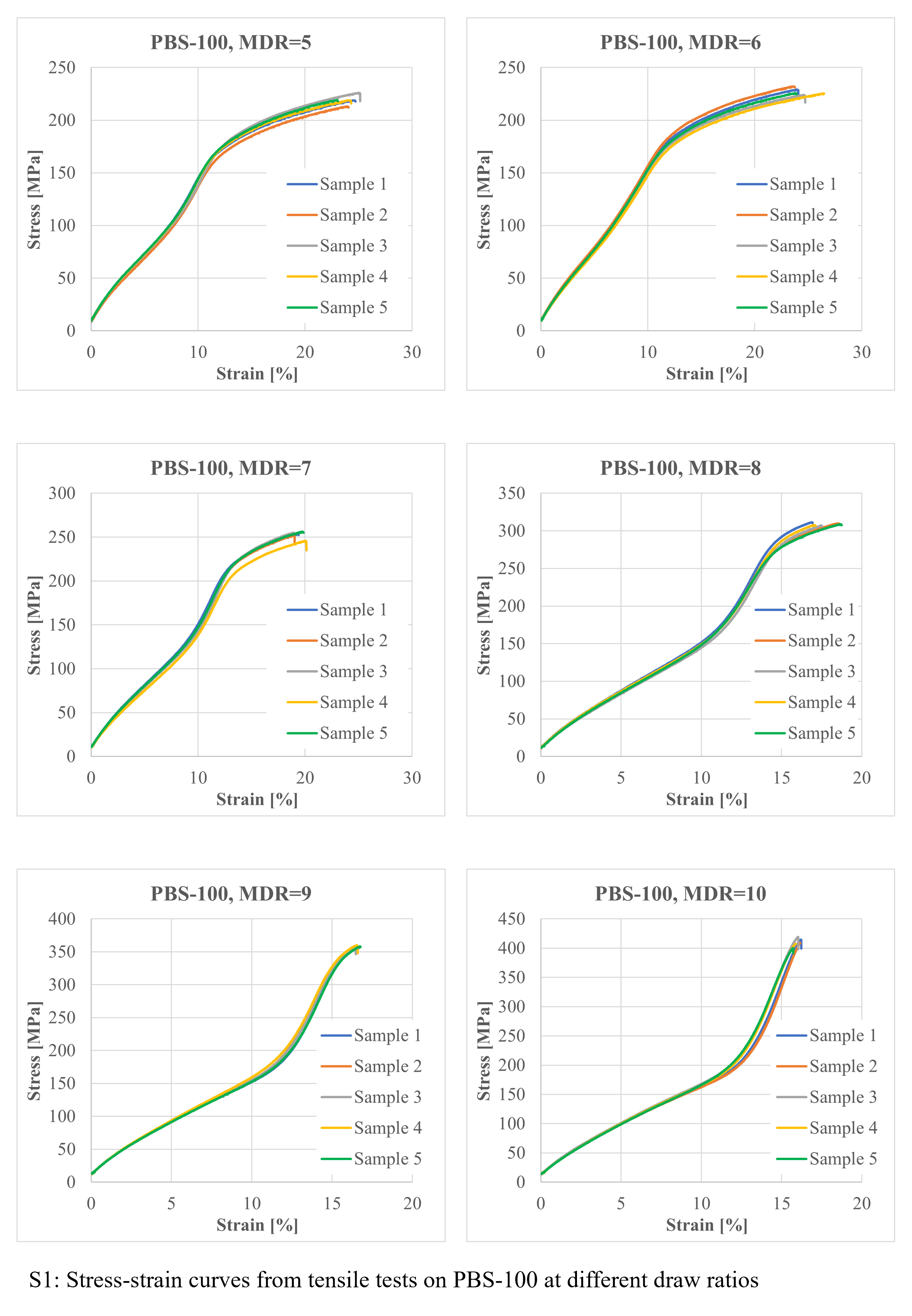

Supplement: Supplementary file 1 [file polymers-17-00403-s001.zip › S1_stress-strain_curves_PBS-100.png]

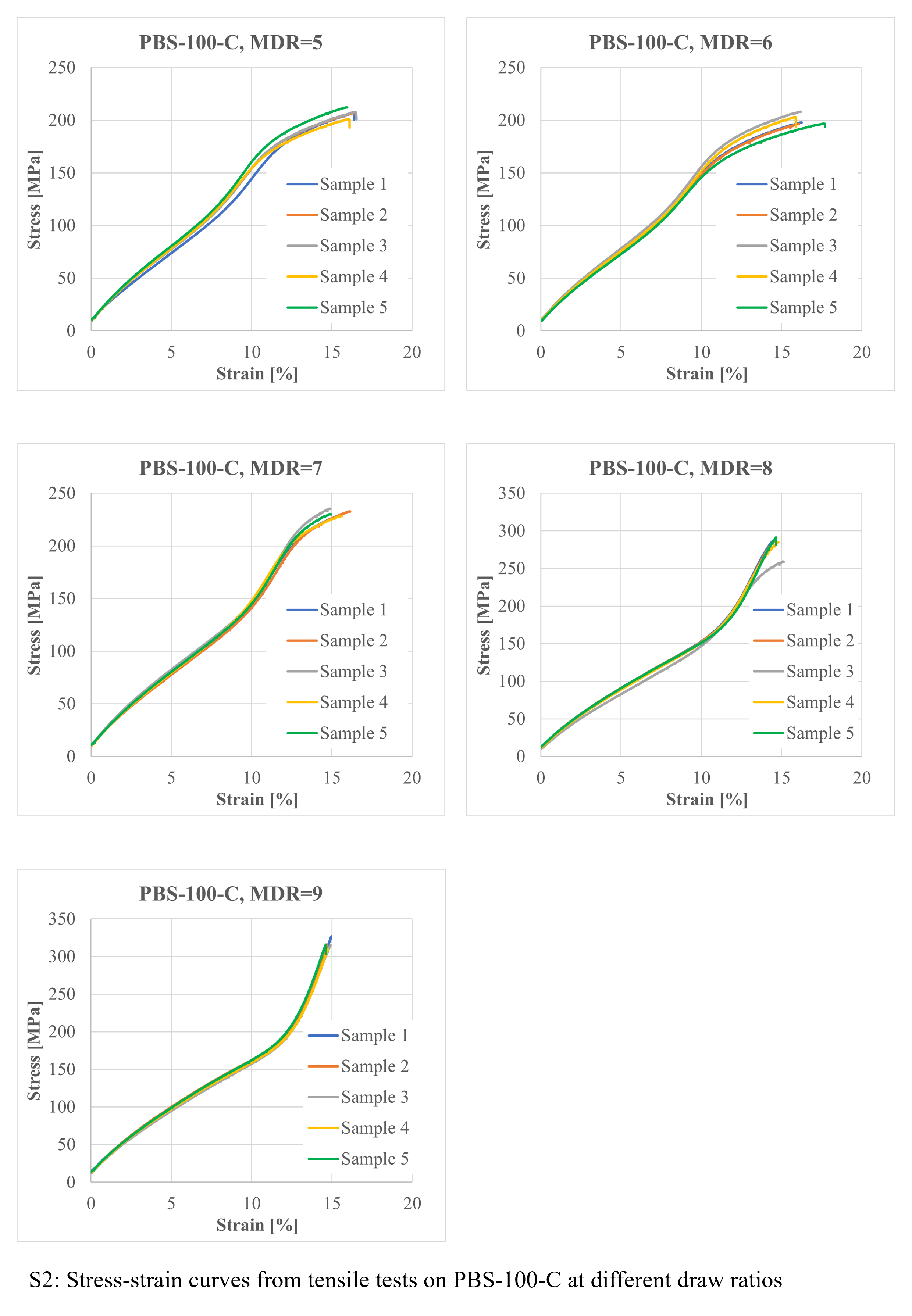

Supplement: Supplementary file 1 [file polymers-17-00403-s001.zip › S2_stress-strain_curves_PBS-100-C.png]

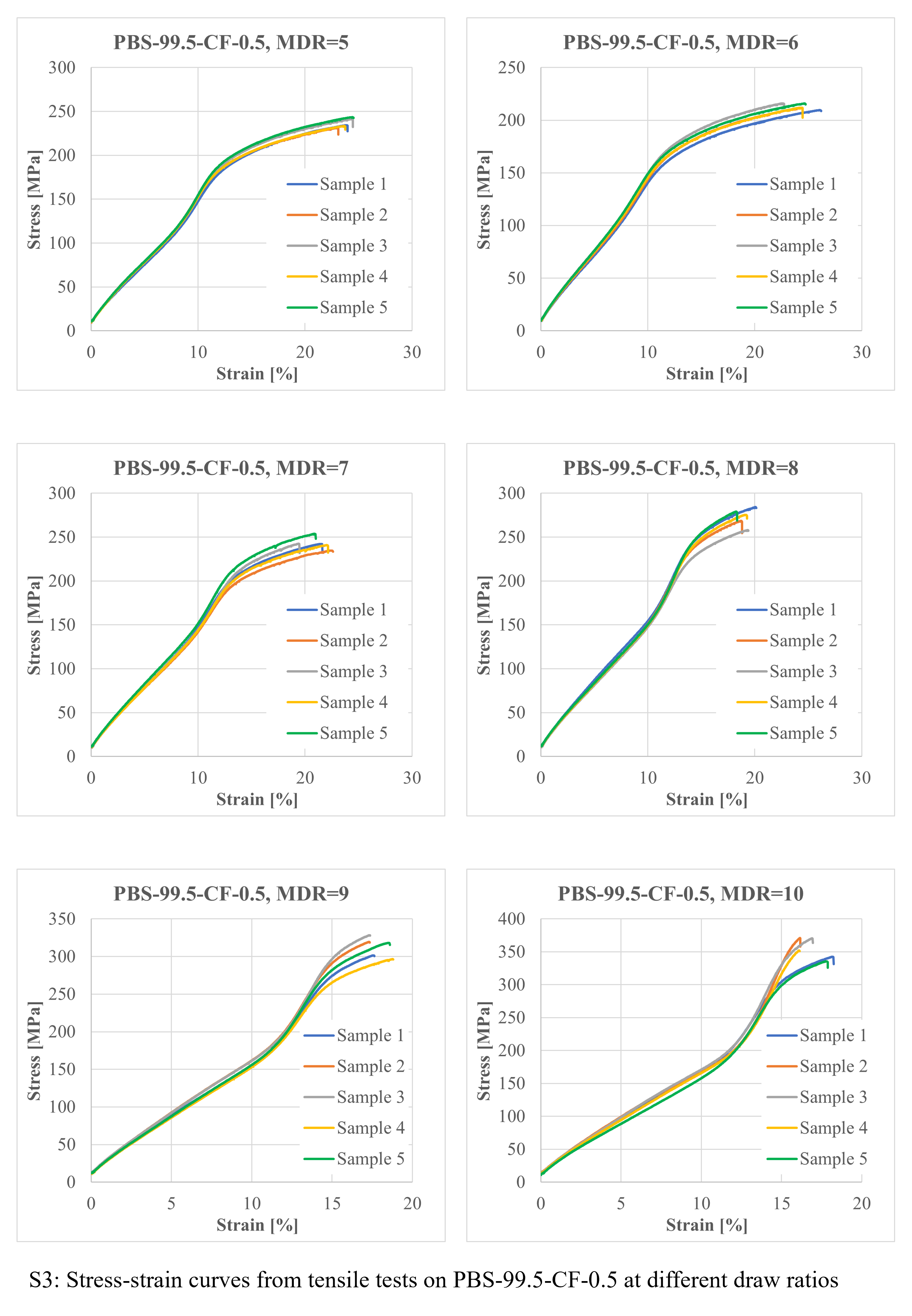

Supplement: Supplementary file 1 [file polymers-17-00403-s001.zip › S3_stress-strain_curves_PBS-99,5-CF-0,5.png]

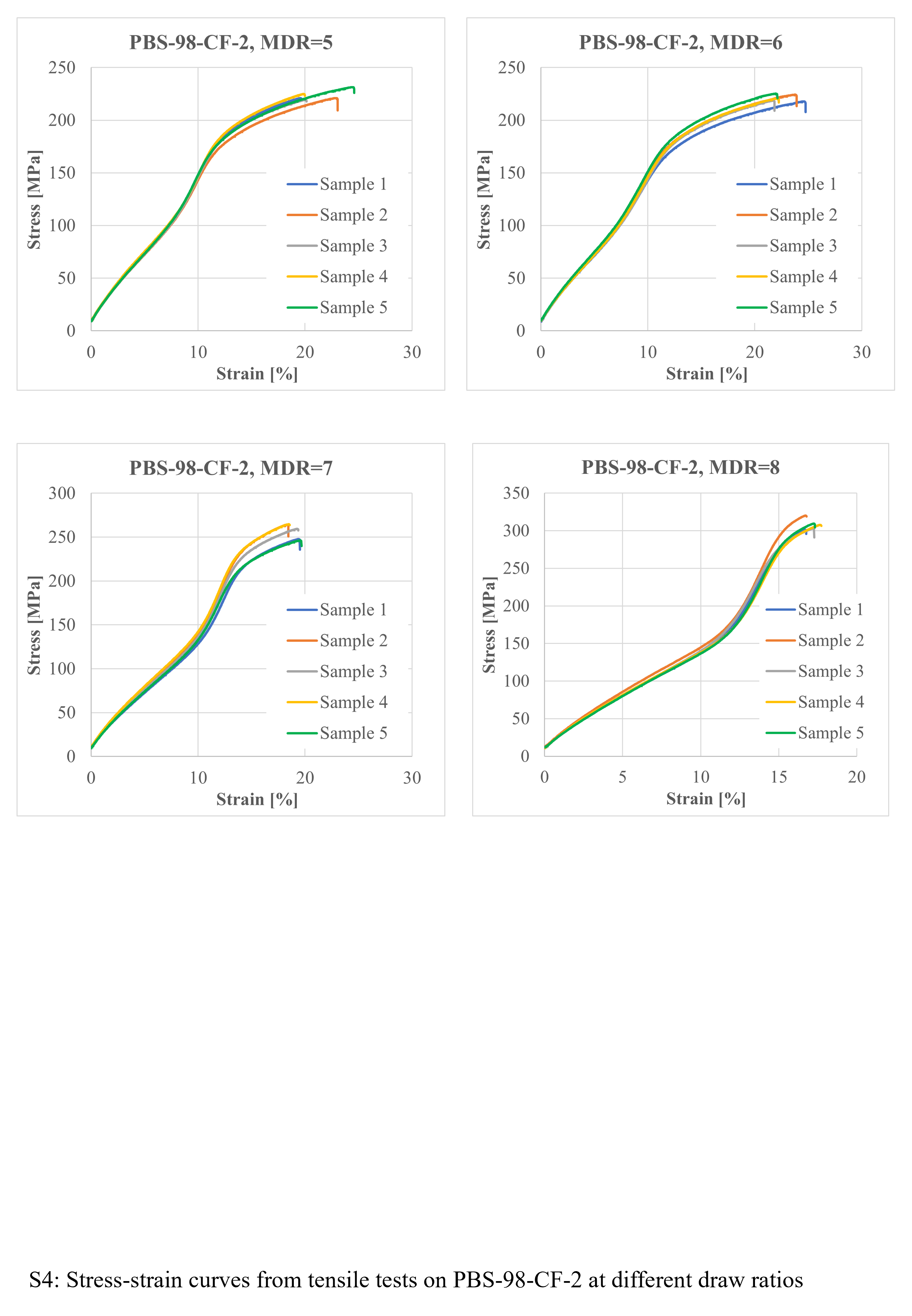

Supplement: Supplementary file 1 [file polymers-17-00403-s001.zip › S4_stress-strain_curves_PBS-98-CF-2.png]

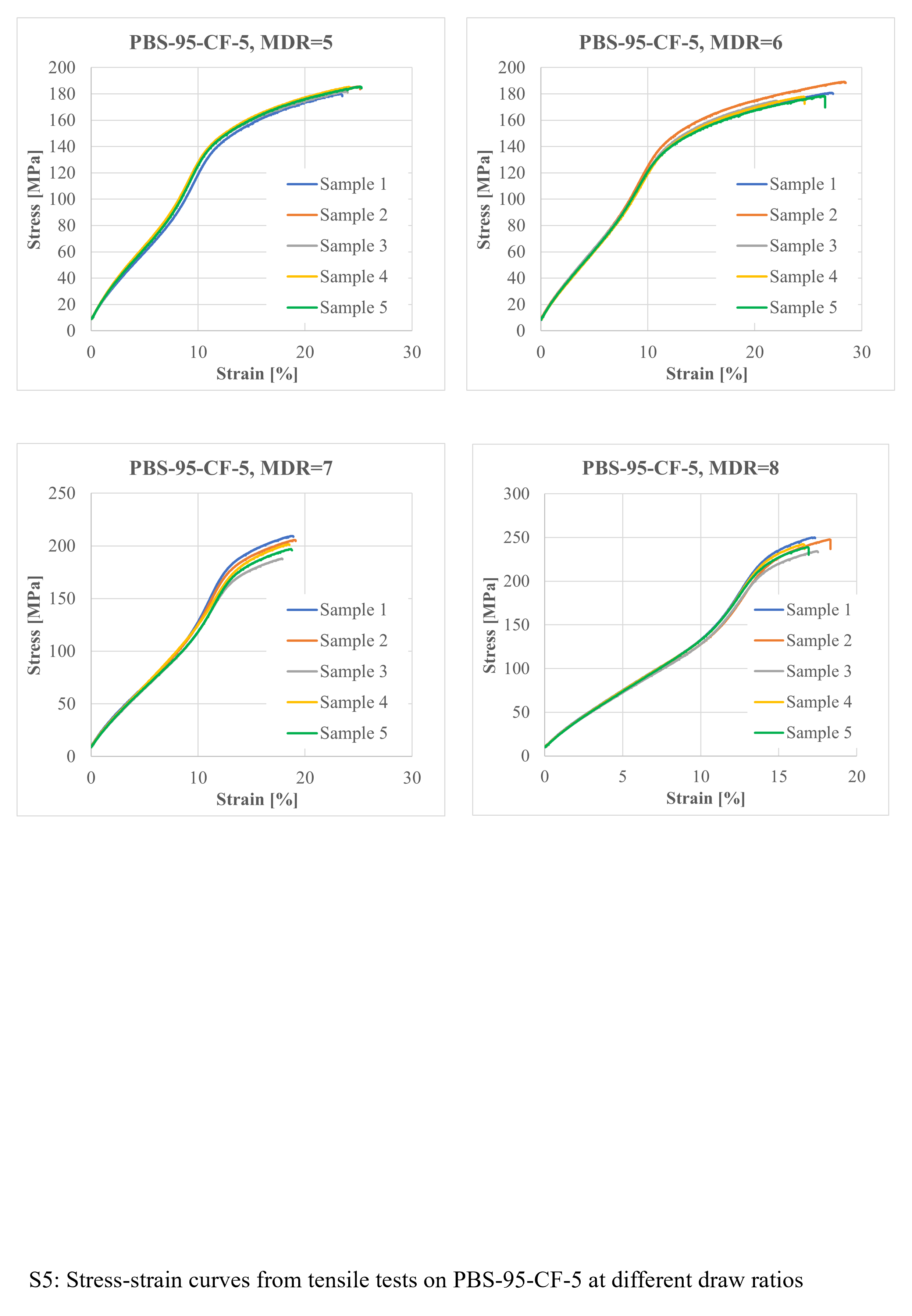

Supplement: Supplementary file 1 [file polymers-17-00403-s001.zip › S5_stress-strain_curves_PBS-95-CF-5.png]

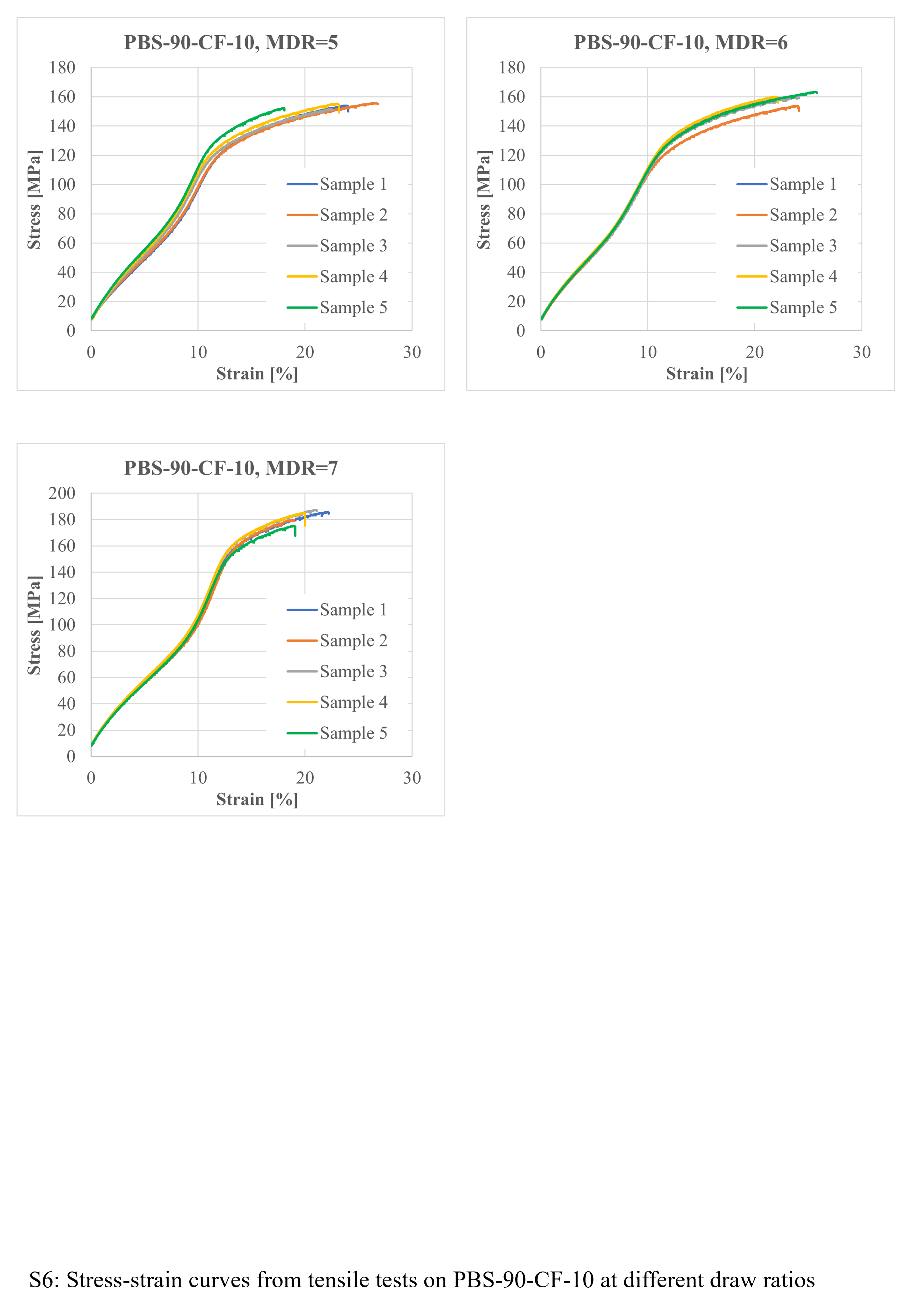

Supplement: Supplementary file 1 [file polymers-17-00403-s001.zip › S6_stress-strain_curves_PBS-90-CF-10.png]

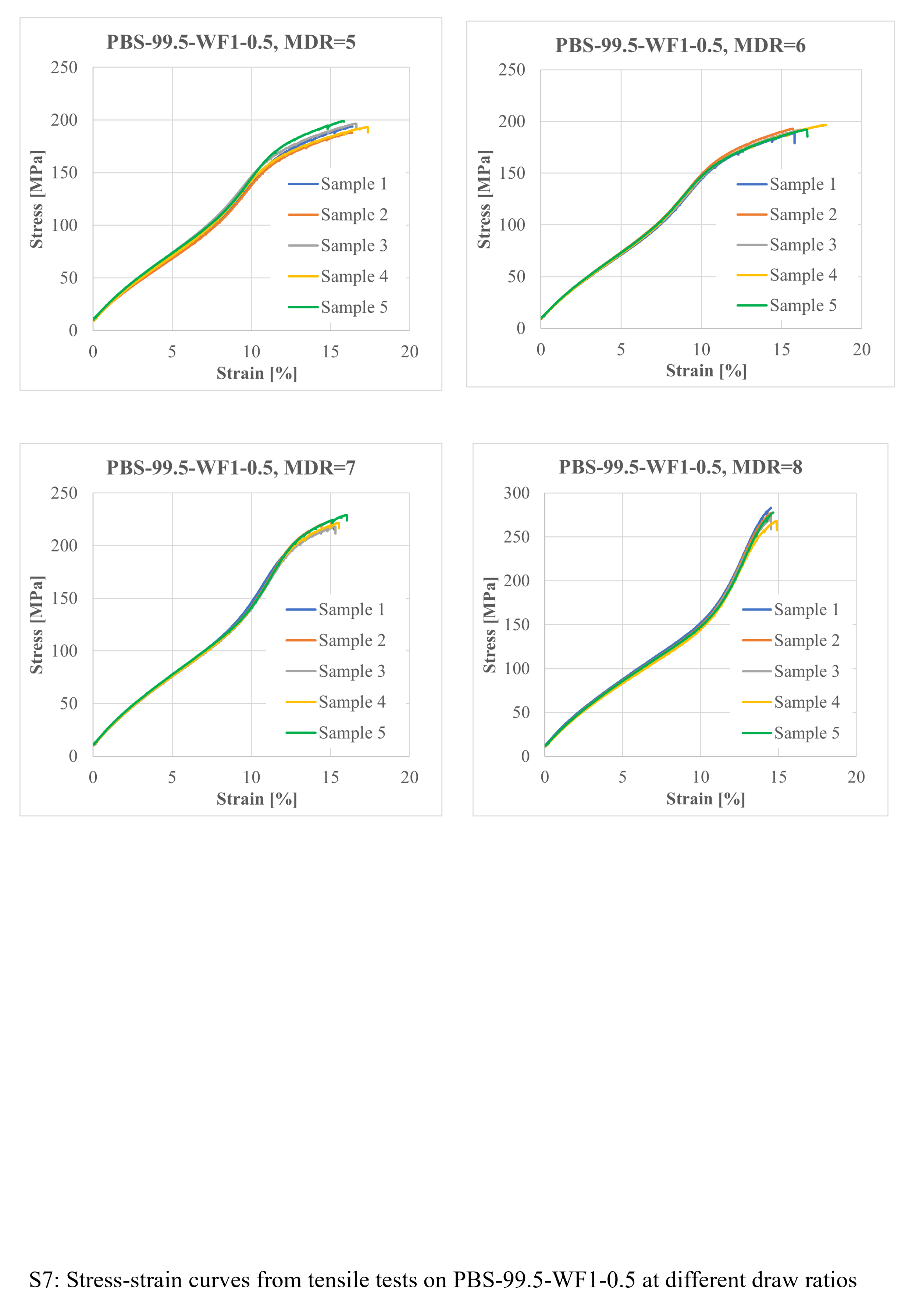

Supplement: Supplementary file 1 [file polymers-17-00403-s001.zip › S7_stress-strain_curves_PBS-99,5-WF1-0,5.png]

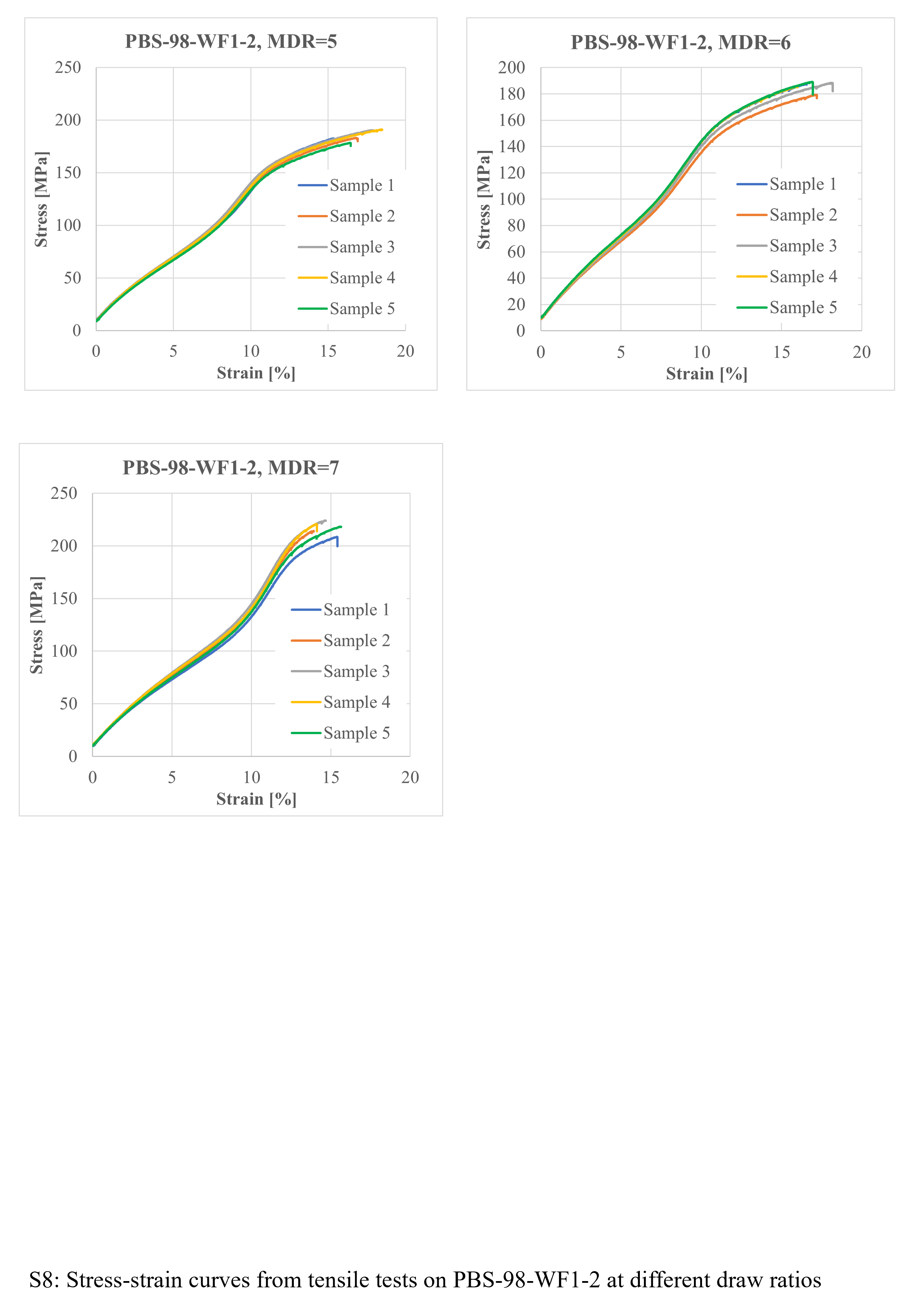

Supplement: Supplementary file 1 [file polymers-17-00403-s001.zip › S8_stress-strain_curves_PBS-98-WF1-2.png]

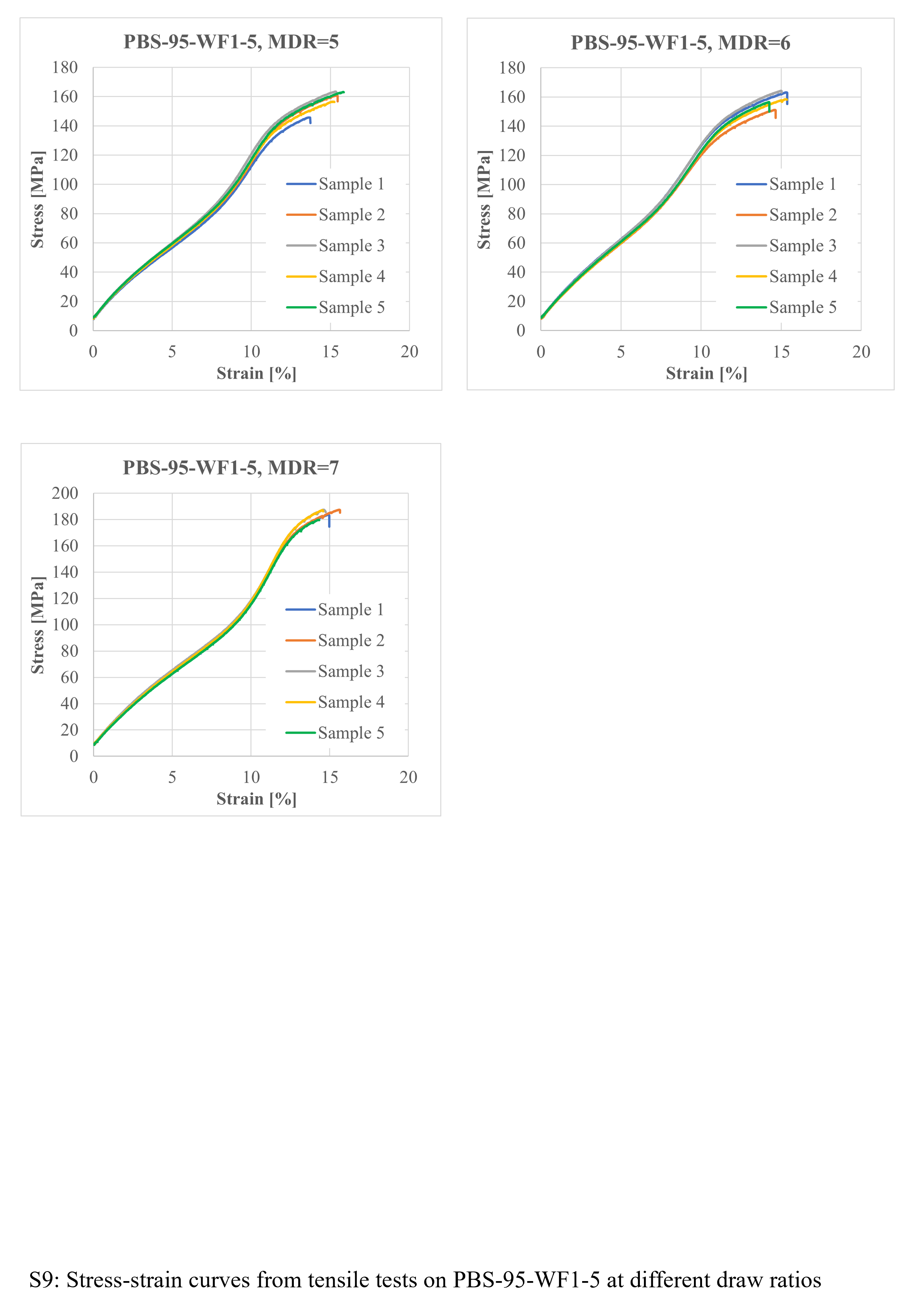

Supplement: Supplementary file 1 [file polymers-17-00403-s001.zip › S9_stress-strain_curves_PBS-95-WF1-5.png]
